# Supplementary material for: Retinal biological age correlates with bone mineral density and fracture risk score and predicts incident osteoporosis
Source: PLOS Digit Health. 2026 May 14;5(5):e0001360. doi: 10.1371/journal.pdig.0001360 (PMC13175334; doi:10.1371/journal.pdig.0001360)
Supplement: S1 Table — (DOCX) [file pdig.0001360.s001.docx]

**S1 Table. Variables field IDs and coding extracted from the UK Biobank**

| Variable names | Data-fields | Coding |
| --- | --- | --- |
| Retinal photos | 21015, 21016 | NA |
| Treatment/medication | 20003 | prednisone  1140868364  methylprednisolone 1140874976  triamcinolone  1140883058 1140883060 1140883062 1140883064 1140868426  dexamethasone  1140874816 1141167174 1141174548  Budesonide  1141174512 1141195232 1141195280 1140862572  prednisolone  1140874930 1140883026 1141157402  hydrocortisone  1140874896 1140876456 1140878562 1140879922 1140879934 1140882822 1140882824 1140882830 1140882836 1140882840 1140882842 1140882844 1140882846 1140882848 1140882850 1140882864 1140882888 1140882894 1140882896 1140882902 1140882906 1140882908 1140882914 1140882918 1140888134 1140910424 1140910634 1141157294 1141181062 1141181610 1141189464 1141194840  betamethasone  1140874790 1140882622 1140882764 1140882766 1140882774 1140882780 1141179982  Cortisone  1140884704 1141173346  deflazacort  1141145782 |
| Had menopause | 2724 | NA |
| ICD diagnosis for systemic diseases | 41270, 41271 | Osteoporosis  M80 M81 M82  Diabetes  E10 – E14  Hypertension  I10 – I15 |
| Ever used hormone-replacement therapy | 2814 | NA |
| MET minutes per week for moderate activity | 22038 | NA |
| MET minutes per week for walking | 22037 | NA |
| Smoking status | 20116 | NA |
| Medical conditions | 2966, 2976 | NA |
| Body mass index | 21001 | NA |
| ICD diagnosis for ocular conditions potentially affecting retinal appearance | 41270, 41271 | Retinal & choroidal disorders  H30, H31, H32, H34, H35, H36  Retinal detachment & breaks  H33  Glaucoma & optic nerve disorders  H40, H42, H46, H47, H48  Lens opacity  H25, H26, H27, H28  Vitreous disorders  H43, H44, H45  Anterior segment disoders  H15, H16, H17, H18, H19, H20, H21, H22  Post-procedure eye  H59 |
| Cataract surgery, self-reported, ICD diagnosis and OPCS-4 code | 5324, 41270, 41272 | Any cataract surgery, including “Right eye only”, “Left eye only”, and “Both eyes”  Vitreous syndrome following cataract surgery  H59.0  Surgery involving lens  C71, C72, C75 |
