# Supplementary material for: Retinal biological age correlates with bone mineral density and fracture risk score and predicts incident osteoporosis
Source: PLOS Digit Health. 2026 May 14;5(5):e0001360. doi: 10.1371/journal.pdig.0001360 (PMC13175334; doi:10.1371/journal.pdig.0001360)
Supplement: S2 Table — (DOCX) [file pdig.0001360.s002.docx]

| **S2 Table. Performance for predicting osteoporotic fracture risk using the Osteoporosis Self-assessment Tool (OST) with and without RetiAGE enhancement.** | | | | | | | | | | |
| --- | --- | --- | --- | --- | --- | --- | --- | --- | --- | --- |
|  | Without RetiAGE | | | |  | With RetiAGE | | | |  |
|  | HR | 95% CI | *p* | C-index |  | HR | 95% CI | *p* | C-index |  |
| OST levels ^a^ | 2.40 | 2.14-2.69 | <0.001 ^c^ | 0.585 |  | 2.09 | 1.86-2.35 | <0.001 ^c^ | 0.635 |  |
| RetiAGE ^b^ | - | - | - | - |  | 1.32 | 1.25-1.40 | <0.001 ^c^ | 0.635 |  |
| HR, hazard ratio; 95% CI, confidence interval; C-index, concordance index. OST, Osteoporosis Self-assessment Tool.  Likelihood ratio test (LRT) between with and without RetiAGE model was significant with the LRT χ² = 88.30, and *p* < 2.20E-16.  ^a^ OST levels of low, Intermediate, and high risk according to published formulas specified for men and women.  ^b^ RetiAGE score was transformed into standardized z-scores, varying from -3 to +3.  ^c^ Statistically significant difference at *p* < 0.05. | | | | | | | | | | |
