# Supplementary material for: Retinal biological age correlates with bone mineral density and fracture risk score and predicts incident osteoporosis
Source: PLOS Digit Health. 2026 May 14;5(5):e0001360. doi: 10.1371/journal.pdig.0001360 (PMC13175334; doi:10.1371/journal.pdig.0001360)
Supplement: S3 Table — (DOCX) [file pdig.0001360.s003.docx]

| **S3 Table. Association between RetiAGE and risk of osteoporosis during follow-up after adjustment for age and gender.** | | | |
| --- | --- | --- | --- |
|  | HR | 95% CI | *p* |
| RetiAGE (continuous)^a^ | 1.12 | 1.05-1.19 | <0.001 ^b^ |
| RetiAGE (quartile) |  |  |  |
| Quartile 1 | 1.00 | [Reference] | - |
| Quartile 2 | 1.15 | 0.96-1.38 | 0.139 |
| Quartile 3 | 1.23 | 1.02-1.49 | 0.028 ^b^ |
| Quartile 4^c^ | 1.36 | 1.12-1.65 | 0.002 ^b^ |
| Age, year | 1.10 | 1.08-1.10 | <0.001 ^b^ |
| Gender ^d^ | 0.18 | 0.16-0.21 | <0.001 ^b^ |
| HR, hazard ratio; 95% CI, confidence interval.  Age and gender were adjusted in the anlysis.  C-index = 0.767, standard error = 0.005.  ^a^ RetiAGE score was transformed into standardized z-scores, varying from -3 to +3.  ^b^ Statistically significant difference at *p* < 0.05.  ^c^ *p* for trend is <0.001.  ^d^ Gender is modeled with women as the reference category. | | | |
