# Supplementary material for: Retinal biological age correlates with bone mineral density and fracture risk score and predicts incident osteoporosis
Source: PLOS Digit Health. 2026 May 14;5(5):e0001360. doi: 10.1371/journal.pdig.0001360 (PMC13175334; doi:10.1371/journal.pdig.0001360)
Supplement: S4 Table — (DOCX) [file pdig.0001360.s004.docx]

**S4 Table. Sensitivity analysis of the association between RetiAGE and osteoporosis in participants without major ocular diseases affecting fundus appearance in the cross-sectional PIONEER cohort.**

|  | OR | 95%CI | *p* |
| --- | --- | --- | --- |
| RetiAGE ^b^ | 1.21 | 1.01-1.46 | 0.037 ^d^ |
| Age, year | 1.01 | 0.98-1.04 | 0.359 |
| Gender ^c^ | 3.13 | 2.13-4.76 | <0.001^d^ |
| Weight, kg | 0.92 | 0.90-0.93 | <0.001^d^ |
| Calcium intake, mg/day | 1.00 | 1.00-1.00 | 0.392 |
| Diabetes | 0.89 | 0.61-1.29 | 0.539 |
| Hypertension | 1.11 | 0.79-1.54 | 0.541 |
| Currently smoking | 0.85 | 0.56-1.29 | 0.449 |
| Light activity, hrs/week | 0.99 | 0.98-1.00 | 0.248 |
| Moderate activity, hrs/week | 0.99 | 0.97-1.01 | 0.267 |
| Glucocorticoids | 0.48 | 0.15-1.56 | 0.224 |
| OR, odds ratio. 95 % CI, 95% confidence interval.  Age, gender, weight, calcium intake, diabetes, hypertension, smoking status, light and moderate activity(hours/week), and ghlucocorticoids were adjusted in the analysis.  Major blinding fundus diseases includes XXX, XXX..  ^a^ Osteoporosis was defined according to WHO guideline of femur neck T-score less than -2.5.  ^b^ RetiAGE was transformed into standardized z-scores, varying from -3 to +3.  ^c^ Gender is modeled with men as the reference category.  ^d^ Statistically significant difference at *p* < 0.05. | | | |
