# Supplementary material for: Retinal biological age correlates with bone mineral density and fracture risk score and predicts incident osteoporosis
Source: PLOS Digit Health. 2026 May 14;5(5):e0001360. doi: 10.1371/journal.pdig.0001360 (PMC13175334; doi:10.1371/journal.pdig.0001360)
Supplement: S6 Table — (DOCX) [file pdig.0001360.s006.docx]

| **S6 Table. Sensitivity analysis of the association between RetiAGE and risk of osteoporosis in participants without ocular conditions potentially affecting retinal appearance in the prospective UK Biobank cohort.** | | | |
| --- | --- | --- | --- |
|  | HR | 95% CI | *p* |
| RetiAGE ^a^ | 1.12 | 1.03-1.21 | 0.009 ^b^ |
| Age, year | 1.10 | 1.09-1.11 | <0.001 ^b^ |
| Gender ^c^ | 5.26 | 4.17-6.25 | <0.001 ^b^ |
| BMI, kg/m^2^ | 0.93 | 0.91-0.95 | <0.001 ^b^ |
| DM history | 1.29 | 0.87-1.77 | 0.202 |
| HTN history | 1.09 | 0.92-1.30 | 0.342 |
| Current smoking | 1.16 | 1.01-1.33 | 0.036 ^b^ |
| MET (moderate), mins/week | 1.00 | 0.99-1.00 | 0.483 |
| MET (walking), mins/week | 1.00 | 0.99-1.00 | 0.363 |
| HR, hazard ratio; 95% CI, confidence interval; BMI, body mass index; DM, diabetes mellitus; HTN, hypertension; MET, metabolic equivalent, measured in minutes.  Age, gender, BMI, diabetes, hypertension, smoking status, and METs of walking and moderate activity(minutes/week) were adjusted in the analysis.  ^a^ RetiAGE score was transformed into standardized z-scores, varying from -3 to +3.  ^b^ Statistically significant difference at *p* < 0.05  ^c^ Gender is modeled with men as the reference category. | | | |
