# Supplementary material for: Retinal biological age correlates with bone mineral density and fracture risk score and predicts incident osteoporosis
Source: PLOS Digit Health. 2026 May 14;5(5):e0001360. doi: 10.1371/journal.pdig.0001360 (PMC13175334; doi:10.1371/journal.pdig.0001360)
Supplement: S9 Table — (DOCX) [file pdig.0001360.s009.docx]

| **S9 Table. Summary of the genome-wide analysis of the RetiAGE score and the implicated genes.** | | | | | | | | |
| --- | --- | --- | --- | --- | --- | --- | --- | --- |
| CHR | SNP | A1FREQ | MAF | BETA | SE | P_BOLT_LMM_INF | Func.refGene | Gene.refGene |
| 2 | rs17713729 | 65.0% | 35.0% | -0.08 | 0.01 | 2.20E-44 | intronic | SH3YL1 |
| 3 | rs9813198 | 51.3% | 48.7% | -0.03 | 0.01 | 3.40E-09 | intronic | CTNNB1 |
| 5 | 5:87846165_GA_G | 92.4% | 7.6% | 0.06 | 0.01 | 4.90E-09 | ncRNA_intronic | LINC00461 |
| 6 | rs12203592 | 79.6% | 20.4% | -0.04 | 0.01 | 4.90E-12 | intronic | IRF4 |
| 6 | rs2395779 | 71.5% | 28.5% | 0.03 | 0.01 | 2.10E-08 | ncRNA_intronic | FOXP4-AS1 |
| 7 | rs10257580 | 99.9% | 0.1% | -0.48 | 0.08 | 8.00E-09 | intergenic | LOC102723427;CT66 |
| 9 | rs11402104 | 73.0% | 27.0% | 0.03 | 0.01 | 1.40E-08 | intergenic | LOC340512;RAD23B |
| 11 | rs321379 | 37.6% | 37.6% | 0.04 | 0.01 | 1.70E-15 | intergenic | ARHGAP20;LINC02550 |
| 12 | rs1919580 | 35.9% | 35.9% | 0.03 | 0.01 | 2.00E-09 | intergenic | PPP1R12A-AS1;OTOGL |
| 14 | rs12891557 | 6.6% | 6.6% | 0.06 | 0.01 | 3.20E-08 | intergenic | MIR4307HG;LOC728755 |
| 15 | rs12913832 | 21.6% | 21.6% | -0.11 | 0.01 | 1.10E-64 | intronic | HERC2 |
| 15 | rs142937747 | 99.1% | 0.9% | 0.22 | 0.04 | 7.40E-09 | exonic | GOLGA8F;GOLGA8G |
| 15 | 15:55502315_CAT_C | 99.9% | 0.1% | -0.69 | 0.12 | 2.80E-08 | intronic | RAB27A |
| 19 | rs778311289 | 99.7% | 0.3% | -0.31 | 0.06 | 3.70E-08 | ncRNA_intronic | LINC00663 |
| CHR: Chromosome number; SNP: Variant identifier (rsID or chromosome:position); A1FREQ: Frequency of the effect allele in the study population; MAF: Minor allele frequency; BETA: Effect size estimate for the effect allele (per allele change in the trait); SE: Standard error of the effect size estimate; P_BOLT_LMM: Association p-value from BOLT-LMM; Func.refGene: Functional annotation of the variant based on RefGene (e.g., intronic, intergenic, exonic); Gene.refGene: Nearest or functionally implicated gene according to RefGene annotations.  RetiAGE heritability h2 = 0.137 (SE= 0.014). | | | | | | | | |
