# Supplementary material for: Retinal biological age correlates with bone mineral density and fracture risk score and predicts incident osteoporosis
Source: PLOS Digit Health. 2026 May 14;5(5):e0001360. doi: 10.1371/journal.pdig.0001360 (PMC13175334; doi:10.1371/journal.pdig.0001360)
Supplement: S10 Table — (DOCX) [file pdig.0001360.s010.docx]

**S10 Table. Distribution of eye diseases and conditions that may affect retinal imaging.**

| Eye conditions | Number of cases | Prevalence |
| --- | --- | --- |
| UK Biobank |  |  |
| Retinal & choroidal disorders | 1,282 | 2.9% |
| Retinal detachment & breaks | 506 | 1.2% |
| Glaucoma & optic nerve disorders | 1,161 | 2.6% |
| Lens opacity | 5,012 | 11.4% |
| Vitreous disorders | 339 | 0.8% |
| Anterior segment disorders | 542 | 1.2% |
| Post-procedure eye | 46 | 0.1% |
| PIONEER |  |  |
| AMD | 164 | 8.4% |
| DR | 186 | 9.5% |
| Glaucoma | 136 | 6.9% |
| The total number of participants included in the prospective UK Biobank cohort is 43,938. The total number of participants included in the cross-sectional PIONEER cohort is 1,950. | | |
