# Supplementary material for: Retinal biological age correlates with bone mineral density and fracture risk score and predicts incident osteoporosis
Source: PLOS Digit Health. 2026 May 14;5(5):e0001360. doi: 10.1371/journal.pdig.0001360 (PMC13175334; doi:10.1371/journal.pdig.0001360)
Supplement: S11 Table — (DOCX) [file pdig.0001360.s011.docx]

**S11 Table. Associations between osteoporosis risk factors and RetiAGE z-score in the cross-sectional PIONEER study.**

| Osteoporosis Risk factors | *β* coefficients ^a^ | Std.error | *P* |
| --- | --- | --- | --- |
| Age | 7.72E-02 | 2.70E-03 | <0.001 ^c^ |
| Gender ^b^ | -1.31E-01 | 4.77E-02 | 0.009 ^c^ |
| Weight | 5.86E-06 | 1.54E-03 | 0.718 |
| Calcium intake, mg/day | -1.27E-04 | 7.26E-05 | 0.092 |
| DM history | 6.59E-02 | 4.41E-02 | 0.159 |
| HTN history | 3.40E-02 | 4.01E-02 | 0.419 |
| Current smoking | 6.54E-02 | 5.17E-02 | 0.225 |
| Light activity, hrs/week | 8.11E-04 | 1.12E-03 | 0.493 |
| Moderate activity, hrs/week | 1.83E-03 | 2.18E-03 | 0.448 |
| Glucocorticoids | 5.82E-02 | 1.31E-01 | 0.656 |
| Std.error, standard error; DM, diabetes mellitus; HTN, hypertension.  ^a^ RetiAGE was the outcome in this analysis, and was transformed into standardized z-scores, varying from -3 to +3.  ^b^ Gender is modeled with men as the reference category.  ^c^ Statistically significant difference at *p* < 0.05. | | | |
