# Supplementary material for: Retinal biological age correlates with bone mineral density and fracture risk score and predicts incident osteoporosis
Source: PLOS Digit Health. 2026 May 14;5(5):e0001360. doi: 10.1371/journal.pdig.0001360 (PMC13175334; doi:10.1371/journal.pdig.0001360)
Supplement: S12 Table — (DOCX) [file pdig.0001360.s012.docx]

**S12 Table. Associations between osteoporosis risk factors and RetiAGE z-score in the prospective UK Biobank cohort.**

| Osteoporosis Risk factors | *β* coefficients ^a^ | Std.error | *p* |
| --- | --- | --- | --- |
| Age | 6.92E-02 | 5.35E-04 | <0.001 ^c^ |
| Gender ^b^ | -5.51E-03 | 8.65E-03 | 0.524 |
| BMI, kg/m^2^ | 5.30E-03 | 9.67E-04 | <0.001 ^c^ |
| DM history | 2.90E-01 | 2.13E-02 | <0.001 ^c^ |
| HTN history | 5.31E-02 | 1.06E-02 | <0.001 ^c^ |
| Current smoking | 6.05E-02 | 8.74E-03 | <0.001 ^c^ |
| MET (moderate), mins/week | 3.78E-06 | 3.96E-06 | 0.340 |
| MET (walking), mins/week | -7.52E-06 | 4.52E-06 | 0.096 |
| Std.error, standard error; DM, diabetes mellitus; HTN, hypertension; MET, metabolic equivalent of task.  ^a^ RetiAGE was the outcome in this analysis, and was transformed into standardized z-scores, varying from -3 to +3.  ^b^ Gender is modeled with men as the reference category.  ^c^ Statistically significant difference at *p* < 0.05. | | | |
