# Supplementary material for: Retinal biological age correlates with bone mineral density and fracture risk score and predicts incident osteoporosis
Source: PLOS Digit Health. 2026 May 14;5(5):e0001360. doi: 10.1371/journal.pdig.0001360 (PMC13175334; doi:10.1371/journal.pdig.0001360)
Supplement: S13 Table — (DOCX) [file pdig.0001360.s013.docx]

**S13 Table. Characteristics of the development set and internal test set of the Korean Health Screening Study.**

|  | Development set | Internal test set |
| --- | --- | --- |
| No. of photos | 129236 | 32318 |
| No. of examinations | 64618 | 16159 |
| No. participants | 40480 | 10171 |
| Age, mean (SD) | 54.0 (9.01) | 53.8 (9.00) |
| Age group, n (%) |  |  |
| 40-49 | 22616 (35.0) | 5623 (34.8) |
| 50-59 | 25525 (39.5) | 6512 (40.3) |
| 60-69 | 12471 (19.3) | 3038 (18.8) |
| 70-79 | 3554 (5.5) | 808 (5.5) |
| ≥80 | 452 (0.6) | 178 (0.6) |

Data are presented as n (% of participants) and mean (standard deviation [SD]).
