# Supplementary figures and images for: Retinal biological age correlates with bone mineral density and fracture risk score and predicts incident osteoporosis
Source: PLOS Digit Health. 2026 May 14;5(5):e0001360. doi: 10.1371/journal.pdig.0001360 (PMC13175334; doi:10.1371/journal.pdig.0001360)

**S1 Fig. Study population flowchart.
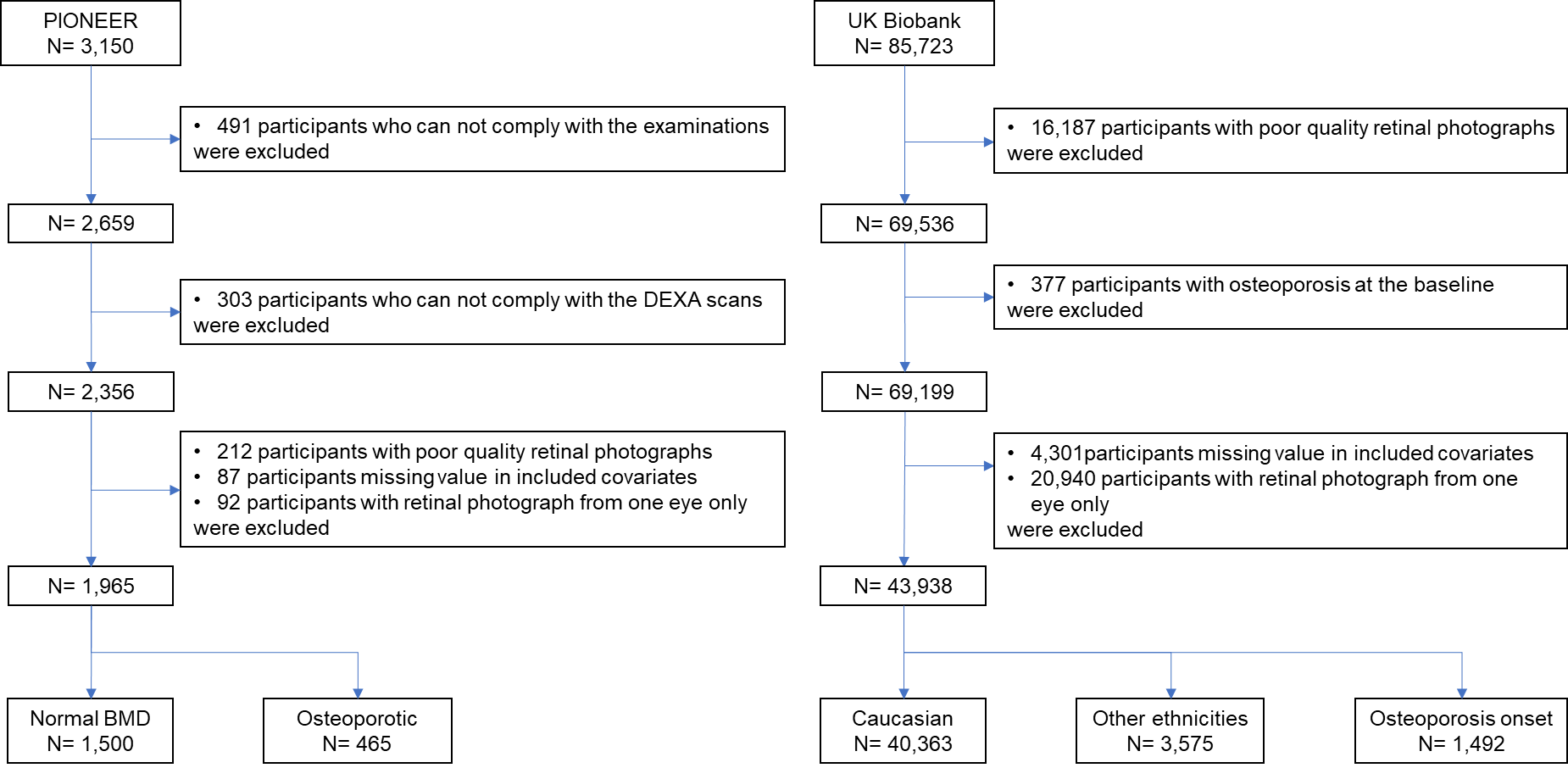
**

Supplement: S1 Fig — (DOCX) [file pdig.0001360.s014.docx]
