# Supplementary material for: Retinal biological age correlates with bone mineral density and fracture risk score and predicts incident osteoporosis
Source: PLOS Digit Health. 2026 May 14;5(5):e0001360. doi: 10.1371/journal.pdig.0001360 (PMC13175334; doi:10.1371/journal.pdig.0001360)
Supplement: S2 Fig — (DOCX) [file pdig.0001360.s015.docx]

**S2 Fig. Association of the retinal biological age marker with bone mineral density and T-score in univariate linear models.**

**Figure Legend**: A. RetiAGE score’s association with bone mineral density among areas; B. RetiAGE score’s association with T-scores among areas. L, lumbar vertebra. Ward’s troch, inter and neck are all regions of the hip bone.
