# Supplementary material for: Retinal biological age correlates with bone mineral density and fracture risk score and predicts incident osteoporosis
Source: PLOS Digit Health. 2026 May 14;5(5):e0001360. doi: 10.1371/journal.pdig.0001360 (PMC13175334; doi:10.1371/journal.pdig.0001360)

**S3 Fig. Net reclassification improvement estimation for osteoporosis risk at 10 years of follow-up.**


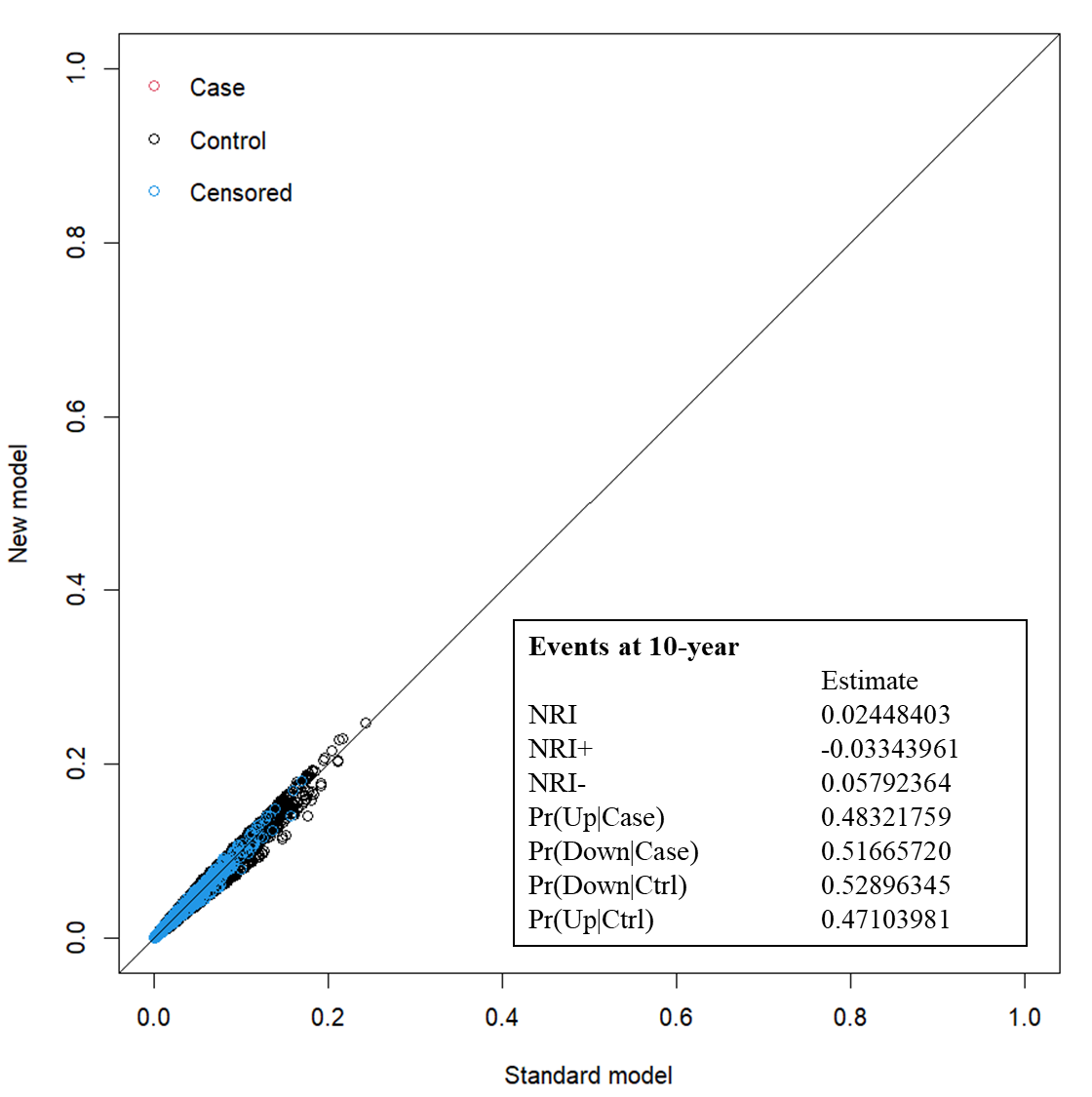

Supplement: S3 Fig — (DOCX) [file pdig.0001360.s016.docx]
