# Supplementary material for: Retinal biological age correlates with bone mineral density and fracture risk score and predicts incident osteoporosis
Source: PLOS Digit Health. 2026 May 14;5(5):e0001360. doi: 10.1371/journal.pdig.0001360 (PMC13175334; doi:10.1371/journal.pdig.0001360)
Supplement: Attachment — Submitted filename: response letter_submitted_version20260106.docx [file pdig.0001360.s019.docx]

Additional Editor Comments (if provided):

The cost of retinal photographs is comparable to that of dual-energy X-ray absorptiometry (DXA). Moreover, DXA involves only minimal radiation exposure and remains the gold standard for assessing bone mineral density (BMD) and diagnosing osteoporosis. Therefore, if retinal photographs merely confirm the association that “accelerated retinal biological aging is associated with decreased BMD and an increased risk of osteoporosis and related fractures,” this finding alone is not sufficiently compelling.

**Response:** Thank you for raising these important and constructive points regarding the clinical relevance of retinal biological age in the context of osteoporosis assessment.

We fully agree that dual-energy X-ray absorptiometry (DEXA) remains the gold standard for assessing bone mineral density (BMD) and diagnosing osteoporosis. However, the cost of retinal photographs is very different from DEXA. In real-world healthcare settings, **the cost, accessibility, and timing of DEXA differ substantially from those of retinal photography**, particularly in the context of population-level screening and early risk stratification.

In Singapore, a DEXA scan typically costs **150–250 SGD** in the public healthcare system and often exceeds **300 SGD** when consultation fees are included (sources: [DEXA in Singapore General Hospital](https://www.sgh.com.sg/tests-procedures/bone-mineral-density-scan-dexa); [DEXA scan Singapore](https://healthscreening.sg/dexa-scan-singapore#:~:text=DEXA%20Scan%20Singapore%20Cost%20;%20DEXA%20Body,with%20Doctor%20for%20Interpretation%20of%20Results%2C%20Tele)). Access to DEXA is also restricted: scans are performed only in hospitals or selected specialised centres and generally require a medical referral. Consequently, DEXA is usually reserved for individuals already suspected of having osteoporosis or fracture risk, rather than for early or opportunistic screening.

In contrast, **retinal photography is low-cost, widely accessible, and already embedded in routine care**. Retinal images are commonly acquired in optical shops, community health centres, and primary care clinics participating in national screening programmes. A retinal photograph typically costs 10–30 SGD, and in large-scale initiatives such as Singapore’s Diabetic Eye Screening Programme, retinal images are obtained without additional cost to patients. Importantly, retinal photography can be performed within minutes by trained nurses or technicians, without radiation exposure or specialist supervision, making it feasible for repeated and community-based assessments.

Therefore, while our findings are **not intended to replace the diagnostic role of DEXA**, they demonstrate that retinal imaging represents a **fast, scalable, and cost-efficient modality** that may function as an **early, population-level indicator of skeletal health risk**, particularly for individuals who would otherwise be unlikely to undergo DEXA testing.

However, if retinal photographs—particularly when combined with AI-driven analysis—can yield information not detectable by DXA alone, this would represent a meaningful and potentially valuable advance. For instance, consider Table 3 (which shows that retinal age predicts osteoporosis and the risk of osteoporotic fracture even after adjusting for established risk factors), a critical question then arises: does retinal age offer predictive value for fracture risk beyond that provided by conventional osteoporosis diagnosis based on DXA?

Specifically, do models using DXA-defined osteoporosis and models using retinal age differ in their ability to predict fractures? If so, can retinal age provide better prognostic information? And, most importantly, does this additional information translate into tangible clinical utility—such as improved risk stratification, earlier intervention, or personalized prevention strategies? Addressing these questions is essential to determine whether retinal imaging coupled with AI represents a clinically relevant tool in osteoporosis and fracture risk assessment.

**Response:** We greatly appreciate the Editor’s suggestion to directly compare the prognostic performance of retinal biological age with DEXA-defined osteoporosis for predicting fracture risk. Unfortunately, such a longitudinal comparison is **not currently feasible** with the available data.

In the UK Biobank, DEXA measurements were obtained only in a non-random subset of participants who attended the imaging assessment visit as part of the Imaging Enhancement Programme, which commenced in 2014. As a result, DEXA measurements occurred **several years after baseline retinal photography** (in Year 2006-2010) and were available for only a small fraction of participants. Among individuals with complete retinal imaging, DEXA measurements, covariates, and follow-up fracture data, the sample size (n= 15) was insufficient to support robust comparative or prognostic modelling. We therefore currently lack appropriately powered longitudinal data to directly compare DEXA-defined osteoporosis and retinal biological age in fracture risk prediction.

Importantly, the **intended clinical role of retinal biological age is not to duplicate DEXA-based diagnosis**, but rather to provide **complementary and earlier risk stratification**, particularly in primary care or screening settings where DEXA is not routinely available.

To address the Editor’s question regarding **incremental predictive value**, we therefore evaluated whether retinal biological age (RetiAGE) improves fracture risk prediction beyond an established non-imaging screening tool used in primary care—the Osteoporosis Self-assessment Tool (OST). In the UK Biobank cohort with longitudinal osteoporotic fracture follow-up, OST alone achieved a **C-index of 0.585**, whereas incorporation of RetiAGE improved predictive performance to a **C-index of 0.635** (**Supplementary Table 2**). This improvement suggests that retinal biological age captures risk information not fully accounted for by conventional clinical screening indices.

Taken together, these findings indicate that retinal biological age can provide additional prognostic information than traditional low-cost tool, Its potential clinical value lies in early identification of individuals at elevated fracture risk prior to DEXA referral, enabling earlier lifestyle modification, preventive strategies, or targeted diagnostic evaluation. Our ongoing longitudinal PIONEER-2 cohort, will allow future direct comparisons between retinal biomarkers and DEXA measurements and further evaluation of their combined clinical utility.

Reviewer 1:

General comments:

Abstract:

This is clear and well-structured, but the use of mechanistic phrasing (e.g., positioning retinal aging as an “indicator” of osteoporosis risk) may overstate causality. I suggest softening this language to emphasise that retinal aging is associated with (rather than definitively indicative of) bone outcomes.

**Response:** We thank the reviewer for this helpful suggestion. We have revised the Abstract to soften mechanistic language and emphasize association rather than causality. Specifically, the conclusion now reads: “Accelerated retinal biological aging is associated with decreased BMD and an increased risk of osteoporosis and related fractures. Retinal age may provide a potential alternative for opportunistic risk screening.”

Introduction:

The Introduction provides a good overview of retinal biomarkers and bone health. However, it would benefit from a more balanced framing of retinal age as an emerging biomarker, including a brief acknowledgment that prior evidence linking retinal aging to systemic outcomes (including BMD) is preliminary and largely exploratory.

**Response:** We appreciate this constructive suggestion. We have revised the Introduction to more explicitly acknowledge the preliminary and exploratory nature of prior evidence. Specifically, we added the following text (**page 3, lines 52–55**): “Together, these preliminary findings suggest that shared biological processes may link retinal health and skeletal integrity. However, direct evidence connecting retinal aging to bone mineral density loss remains limited, and few studies have examined whether a homogenous biological aging process underlies changes in both tissues.”

Rows 61–66:

The IRF4 pathway is indeed relevant to bone remodeling and osteoporosis (Wu et al., 2024; Weivoda & Bradley, 2023), and your group has made important contributions to retinal biological age as a biomarker (Nusinovici et al., 2022; Zhu et al., 2022). At present, however, large GWAS of retinal age gap and related traits highlight loci such as ALKAL2, OCA2/HERC2, and POC5 rather than IRF4 (Ahadi et al., 2023; Goallec et al., 2021). This suggests that while the IRF4–bone connection is biologically established, its role in retinal aging is still more inferential. The association between retinal aging and bone outcomes, including osteoporosis, is promising but remains preliminary and mostly reported at an abstract level (Peng et al., 2025, ARVO abstract). To strengthen the rationale, I suggest either (i) reframing this section as a hypothesis or potential biological bridge, or (ii) if the authors believe the IRF4–retina–bone pathway has stronger validation, providing additional evidence or references to support it.

**Response:** We thank the reviewer for this important clarification. We agree that prior large-scale GWAS of retinal aging–related traits have highlighted loci such as *ALKAL2*, *OCA2/HERC2*, and *POC5*, and that evidence linking *IRF4* to retinal aging has previously been limited.

To address this, we conducted a genome-wide association study of RetiAGE in 45,496 White UK Biobank participants after the initial publication of the RetiAGE model. We have added both the GWAS methods and results to the manuscript. IRF4 emerged as one of the genome-wide significant loci (**Supplementary Table 9**). While GWAS findings do not imply causality, they provide genetic support that pathways implicated in bone biology may also be associated with inter-individual variation in retinal biological aging.

We have therefore reframed this section to present the IRF4–retina–bone link as a hypothesis supported by emerging genetic evidence, rather than as an established mechanistic pathway.

In the method section, **page 10, line 252-259**, we added: “*For the GWAS analysis, we included 45,496 White participants in the UK Biobank with readable retinal photos from both eyes. We first calculated the heritability for RetiAGE score. The single nucleotide polymorphism (SNPs) with an imputation quality score R2 below 0.3 and a minor allele frequency (MAF) less than 1% were excluded from the analyses. A linear-mixed model used applied to account for relatedness, as represented by the genetic relationship matrix. The analyses were then conducted using BOLT-LMM software under an infinitesimal mixed model and adjusted for sex, age, genotype array, and up to 20 principal components to account for population substructures within the European population.”*

| **Supplementary table 9. Summary of the genome-wide analysis of the RetiAGE score and the implicated genes.** | | | | | | | | |
| --- | --- | --- | --- | --- | --- | --- | --- | --- |
| CHR | SNP | A1FREQ | MAF | BETA | SE | P_BOLT_LMM | Func.refGene | Gene.refGene |
| 2 | rs17713729 | 65.0% | 35.0% | -0.08 | 0.01 | 2.20E-44 | intronic | SH3YL1 |
| 3 | rs9813198 | 51.3% | 48.7% | -0.03 | 0.01 | 3.40E-09 | intronic | CTNNB1 |
| 5 | 5:87846165_GA_G | 92.4% | 7.6% | 0.06 | 0.01 | 4.90E-09 | ncRNA_intronic | LINC00461 |
| 6 | rs12203592 | 79.6% | 20.4% | -0.04 | 0.01 | 4.90E-12 | intronic | IRF4 |
| 6 | rs2395779 | 71.5% | 28.5% | 0.03 | 0.01 | 2.10E-08 | ncRNA_intronic | FOXP4-AS1 |
| 7 | rs10257580 | 99.9% | 0.1% | -0.48 | 0.08 | 8.00E-09 | intergenic | LOC102723427;CT66 |
| 9 | rs11402104 | 73.0% | 27.0% | 0.03 | 0.01 | 1.40E-08 | intergenic | LOC340512;RAD23B |
| 11 | rs321379 | 37.6% | 37.6% | 0.04 | 0.01 | 1.70E-15 | intergenic | ARHGAP20;LINC02550 |
| 12 | rs1919580 | 35.9% | 35.9% | 0.03 | 0.01 | 2.00E-09 | intergenic | PPP1R12A-AS1;OTOGL |
| 14 | rs12891557 | 6.6% | 6.6% | 0.06 | 0.01 | 3.20E-08 | intergenic | MIR4307HG;LOC728755 |
| 15 | rs12913832 | 21.6% | 21.6% | -0.11 | 0.01 | 1.10E-64 | intronic | HERC2 |
| 15 | rs142937747 | 99.1% | 0.9% | 0.22 | 0.04 | 7.40E-09 | exonic | GOLGA8F;GOLGA8G |
| 15 | 15:55502315_CAT_C | 99.9% | 0.1% | -0.69 | 0.12 | 2.80E-08 | intronic | RAB27A |
| 19 | rs778311289 | 99.7% | 0.3% | -0.31 | 0.06 | 3.70E-08 | ncRNA_intronic | LINC00663 |
| CHR: Chromosome number; SNP: Variant identifier (rsID or chromosome:position); A1FREQ: Frequency of the effect allele in the study population; MAF: Minor allele frequency; BETA: Effect size estimate for the effect allele (per allele change in the trait); SE: Standard error of the effect size estimate; P_BOLT_LMM: Association p-value from BOLT-LMM; Func.refGene: Functional annotation of the variant based on RefGene (e.g., intronic, intergenic, exonic); Gene.refGene: Nearest or functionally implicated gene according to RefGene annotations.  RetiAGE heritability h^2^ = 0.137 (SE= 0.014). | | | | | | | | |

Methods:

The description of the statistical models is relatively brief. Could the authors clarify (i) whether any corrections for multiple comparisons were applied given the number of outcomes tested, (ii) whether interaction terms (e.g., age, sex) were evaluated, and (iii) how missing data were handled in covariates?

**Response:** We thank the reviewer for these helpful methodological questions.

(i) For analyses involving multiple bone mineral density outcomes, we adjusted for multiple comparisons using the false discovery rate (FDR) method. The FDR method was mentioned **on page 7, between line 173-177**, “…and adjusted for multiple testing with False Discovery Rate method (FDR).”

(ii) Because RetiAGE is intrinsically correlated with calendar age, we did not include interaction terms between RetiAGE and age in the primary models to avoid multicollinearity. Instead, we assessed potential effect modification by conducting stratified analyses across age and sex subgroups (**Table 5**, and **Supplementary Table 8**), as now clarified in the Methods.

(iii) Participants with missing values in key covariates were excluded during data preparation, as shown in the **Supplementary figure 1**.

Rows 97–99:

The methods section states that the “macula-centered retinal photograph with the best quality judging by an in-house quality control model” was chosen for each eye, and that scores were averaged across two eyes. This is somewhat vague. Could the authors provide more details on: (i) the criteria or thresholds used by the quality control model, (ii) whether images from both eyes were always available and included, and (iii) how missing or poor-quality images were handled in the averaging process?

**Response:** Thanks for pointing out the lack of information on the in-house quality control model. (i) We added further descriptions **on page 5, line 111-115**: “In each eye, the macula-centered retinal photograph with the best quality judging by an in-house quality control model with a threshold of 0.593, were chosen to generate the RetiAGE scores. The in-house quality control model was trained on over 20,000 quality controlled retinal images with a Swin transformer. The model achieved an AUC of 0.97 classifying the retinal image readability.”

(ii) As the RetiAGE algorithm requires retinal images from both eyes to generate the RetiAGE scores, we only included participants with retinal images available in both eyes. We added a sentence in the exclusion criteria on **page 7, line 160-161**: “The participants with readable retinal photos from only one eye were excluded.” Nevertheless, the difference in the score between individuals’ two eyes are small. To assess potential laterality effects, we additionally evaluated inter-eye agreement in the PIONEER study on **page 5, line 116-117**. The RetiAGE scores demonstrated moderate inter-eye agreement (**ICC = 0.65, 95% CI 0.62–0.68**), with a small mean right–left difference (**mean difference = 0.04, SD = 0.23**), indicating no meaningful systematic laterality bias.

(iii) Images classified as unreadable by the quality control model were excluded and not used for score generation. RetiAGE scores were calculated by averaging scores derived from readable images from both eyes, and no imputation was applied for missing or poor-quality images.

Rows 102–103:

The description of the DEXA scans as using “very low dose X-rays (~0.005 to 0.01 mSv)” could benefit from clarification. Was this range measured in your study, taken from manufacturer specifications, or cited from prior literature? It may be helpful to specify how the dose was determined, and to explain what “very low dose” means (e.g., compared to background radiation or a chest X-ray). Also, the placement of the reference (20) should be integrated into the sentence (currently appears as a typo). The same formatting issue appears elsewhere in the manuscript and may need systematic correction.

**Response:** The authors appreciate this comment. We checked the source of the dosage, and added the manufacturer’s manual to the DEXA description **on page 6, line 120-121**: *“The DEXA scans (Hologic Discovery-W; Hologic Inc, Bedford-MA) were taken with X-rays (~0.005 to 0.01 millisievert, Horizon User Guide MAN-08072-002 Rev 003) for the PIONEER participants.[20]”* We also revised all the citation styles upon suggestion.

Rows 105–106:

The description of T-scores in the Methods is a bit unclear. It currently states that the T-score compares each participant’s BMD to “the peak bone mass of a healthy 30-year-old adult.” Could the authors clarify how this reference value is determined? Is it based on a fixed age (30 years), or on a manufacturer-provided young adult reference database built into the DEXA system?

**Response:** DEXA-derived T-scores were obtained directly from the DEXA system and calculated using manufacturer-provided reference databases based on young-adult populations. Specifically, T-scores were referenced to the Third National Health and Nutrition Examination Survey (NHANES III) young-adult normative database, consistent with World Health Organization diagnostic criteria. To improve clarity, we revised the Methods section (**page 6, lines 123–126**) to read: *“The BMD T-score represents the number of standard deviations by which an individual’s measured BMD differs from the mean BMD of a young-adult reference population, as defined by the manufacturer-provided Third National Health and Nutrition Examination Survey (NHANES III) norms.”*

Rows 113–120:

Given that retinal structure, pigmentation, and vascular patterns can vary across ethnic groups, and that your algorithm was originally developed in a Korean population, it would be helpful if the manuscript could clarify how well the RetiAGE model performs across the different ethnicities represented in PIONEER (Chinese, Indian, Malay) and in the predominantly European UK Biobank cohort. Since you also use FRAX scores, which are themselves calibrated to population-specific fracture and mortality data, it might be worth commenting on how ethnicity could influence both retinal age predictions and the fracture risk assessment.

**Response:** RetiAGE is a retinal biological aging marker for which no direct ground-truth measure of biological age exists. In prior studies, we evaluated its performance by stratifying mortality risk across multiple populations, including Korean and UK Biobank cohorts.

As a probabilistic score derived from retinal images, differences in population characteristics may lead to shifts in absolute score distributions, while having limited impact on discrimination within cohorts. We also observed similar distributions across the Singaporeans. To illustrate this, we have added scatter plots showing the distribution of RetiAGE against calendar age in each study population (**Supplementary Figure 4**).

We now more explicitly acknowledge in the Methods and Discussion that ethnic differences in retinal features and population-specific calibration of FRAX scores may influence absolute risk estimates, and that our analyses focus on within-cohort associations rather than direct cross-population comparisons.

Results:

The Results present associations between RetiAGE and bone outcomes, but the magnitude of effect sizes is not always discussed in terms of clinical relevance. For example, how do the observed differences in BMD or fracture risk compare to known risk factors such as smoking, BMI, or diabetes?

**Response:** We appreciate the reviewer’s comment regarding clinical interpretation of effect sizes. In the cross-sectional PIONEER analyses, older age, female sex, and lower body weight were the traditional risk factors significantly associated with osteoporosis and FRAX-derived fracture risk. These variables constitute the core components of the Osteoporosis Self-assessment Tool (OST), a widely used screening instrument in primary care.

In the prospective UK Biobank analyses, diabetes did not show a significant association with the risk of osteoporosis. Lifestyle factors, including current smoking (HR per SD= 1.07) and lower physical activity (MET of walking, HR per SD= 0.94, inverse HR= 1.06), were associated with osteoporosis risk, but these associations were weaker than those observed for age (HR per SD= 2.23), gender (HR per SD= 2.37), BMI (HR per SD= 0.69, inversed HR per SD= 1.45), and retinal biological age (HR per SD= 1.12). These findings underscore the robustness of OST while motivating evaluation of whether retinal biological age could provide incremental prognostic value. The standardized effect sizes are now reported on **page 11, line 294-299**. The related statistical methods are described in the Method section, on **page 9, line 233-238**.

Accordingly, we assessed whether inclusion of RetiAGE improved fracture risk prediction beyond OST. Likelihood ratio testing (LRT χ² = 88.30, *p* < 2.20E-16, **Supplementary Table 2**) demonstrated improved model performance when RetiAGE was incorporated, supporting its potential role as a complementary, opportunistic risk stratification marker.

We added a few sentences in the discussion about the effect sizes of OST variables and the other risk factors **on page 16, line 423-444**: *“In clinical practice, DEXA is typically reserved for individuals already considered at elevated risk of osteoporosis and is not routinely used as a population-level screening tool due to cost, accessibility, and resource constraints. As a result, osteoporosis screening in primary care often relies on simple risk calculator such as the Osteoporosis Self-assessment Tool (OST), which incorporates calendar age, sex, and body weight. Consistent with this framework, in the cross-sectional PIONEER analyses, higher age, female sex, and lower body weight were the principal non-retinal factors associated with osteoporosis (****Table 3****), underscoring the robustness of OST-based risk stratification. In the prospective UK Biobank analyses, traditional metabolic comorbidities such as diabetes and hypertension were not significantly associated with incident osteoporosis in the multivariable Cox Ph model. Lifestyle factors, including current smoking and lower physical activity, were associated with osteoporosis risk, but these associations were weaker than those observed for demographic factors and retinal biological age. These findings motivated evaluation of whether retinal biological age could provide incremental prognostic value beyond established OST variables. Accordingly, inclusion of RetiAGE significantly improved longitudinal model fit in likelihood ratio testing, indicating enhanced identification of individuals at elevated fracture risk (****Supplementary Table 2****). Taken together, these findings suggest that retinal biological age may capture information related to skeletal aging that is not fully reflected by conventional demographic or lifestyle risk factors. While DEXA remains the standard for diagnosing osteoporosis, retinal biological age may serve as a complementary marker in research or exploratory screening contexts. Given that retinal aging can precede overt ocular disease, retinal imaging may offer a window into systemic aging processes relevant to bone health.”*

Figures and Tables:

Figures are generally clear, though the legends could be more descriptive. For example, specifying the covariates adjusted for in regression models directly in the figure legends would make them easier to interpret without needing to cross-reference the Methods.

**Response:** We thank the reviewer for raising this important point. We have made changes to the table names and footnotes, including explicit listing of covariates adjusted for in each model, to improve the readability.

Discussion:

One area that may warrant further clarification is the role of ethnicity. In the Methods, it would indeed be useful to explain how the RetiAGE algorithm, originally developed in a Korean population, was applied to the PIONEER cohort (Chinese, Indian, Malay) and the predominantly European UK Biobank, and whether any performance differences were observed across groups. Here, in the Discussion, a brief acknowledgment that retinal pigmentation and vascular patterns may vary by ethnicity, and that FRAX scores are themselves calibrated to population-specific fracture and mortality risks, would strengthen this manuscript.

**Response:** We agree with the reviewer that ethnic differences in retinal pigmentation, structure, and vascular patterns, as well as population-specific calibration of FRAX scores, warrant acknowledgment. We have rephrased the study limitation **on page 15-16, line 453-459**: “…*Second, although we observed consistent cross-sectional and prospective associations of retinal biological aging with bone mineral density and osteoporosis-related outcomes across populations differing in ethnicity and calendar age, retinal pigmentation and structure, and vascular patterns may vary among ethnic groups. While FRAX-derived fracture risk scores are calibrated using population-specific data, retinal aging models trained or fine-tuned in specific ethnic populations may further optimize the performance of RetiAge when applied to ethnicity-specific risk estimation.”*

It might be worth briefly mentioning “non-IRF4” pathways that could explain shared biology between retinal and bone aging, such as systemic inflammation, microvascular changes, or oxidative stress.

**Response:** We have also expanded the Discussion to note that pathways beyond IRF4—including systemic inflammation, microvascular dysfunction, and oxidative stress—may contribute to shared retinal and skeletal aging, consistent with gene enrichment findings reported in prior GWAS of retinal aging markers. Please see changes **on page 14, line 408-420**: “*Beyond IRF4-related immune regulation, several systemic aging pathways may plausibly link retinal and skeletal health. Chronic low-grade inflammation, microvascular dysfunction, and oxidative stress are well-recognized contributors to both retinal degeneration and bone mineral loss. These processes can impair retinal perfusion, disrupt neurovascular integrity, and accelerate photoreceptor and microglial dysfunction, while concurrently promoting bone resorption and reducing bone formation. Consistent with this concept, epidemiological studies have reported increased osteoporosis risk among patients with dry eye disease and a higher prevalence of age-related macular degeneration among postmenopausal women with osteoporosis, with impaired immune and inflammatory regulation proposed as shared mechanisms. Supporting a broader biological overlap, gene enrichment analyses from prior GWAS of retinal aging markers have also identified associations with non-ocular pathways, including bone mineralization and systemic metabolic processes.*”

Reviewer 2:

* The manuscript states that RetiAGE reflects the likelihood of being ≥65 years. Please justify the choice of this threshold. Is ≥65 a clinically motivated cut-off, or an artifact of the training data distribution?

**Response:** The authors thank the reviewer for this comment. The ≥65-year threshold reflects a pragmatic and clinically interpretable definition of older age that is widely used in epidemiological and health services research. RetiAGE is used as a continuous probability score in downstream analyses, and results are therefore not entirely dependent on a hard threshold. In our prior work, we evaluated alternative thresholds (65, 70, and 75 years) and observed that shifts in the reference threshold did not materially affect discrimination or downstream associations (**Appendix 4** in [Nusinovici et al., Age Ageing 2022](https://academic.oup.com/ageing/article/51/4/afac065/6561972?login=false)).

In the present study, we used the 65-year reference to facilitate interpretability and consistency across cohorts with differing age distributions (UK Biobank, calendar age 56.2 ± 8.1; PIONEER, calendar age 72.5 ± 8.2, **Table 1**). The distributions of RetiAGE relative to calendar age in both the UK Biobank and PIONEER cohorts are now shown in **Supplementary Figure 4**.

Furthermore, from a public health perspective, age ≥65 years is also commonly used to define older adults in population statistics, including the Singapore Department of Statistics ([National Statistical Standard: Recommendations on Definition and Classification of Age](https://www.singstat.gov.sg/-/media/files/standards_and_classifications/nsa.ashx#:~:text=For%20the%20purpose%20of%20compiling,persons%20aged%2020%20%E2%80%93%2064%20years) ). We have clarified the rationale for this reference age in the Methods section, on **page 4, line 84-85.**

* Provide the age distribution of the training population and the disease distribution (including relevant ophthalmic conditions) to contextualize the biomarker.

**Response:** The age distribution and key characteristics of the RetiAGE development and internal validation cohorts have been reported in detail in the original publication ([Nusinovici et al., Age Ageing 2022](https://academic.oup.com/ageing/article/51/4/afac065/6561972?login=false)) and are reproduced in the **Supplementary Table 13** for completeness. Retinal images were not excluded based on ophthalmic diagnoses unless image quality was insufficient, as age-related ocular changes are considered part of the biological aging signal captured by the model.

**Supplementary Table 13**. Characteristics of the development set and internal test set of the Korean screening health study

|  | Development set | Internal test set |
| --- | --- | --- |
| No. of photos | 129236 | 32318 |
| No. of examinations | 64618 | 16159 |
| No. participants | 40480 | 10171 |
| Age, mean (SD) | 54.0 (9.01) | 53.8 (9.00) |
| Age group, n (%) |  |  |
| 40-49 | 22616 (35.0) | 5623 (34.8) |
| 50-59 | 25525 (39.5) | 6512 (40.3) |
| 60-69 | 12471 (19.3) | 3038 (18.8) |
| 70-79 | 3554 (5.5) | 808 (5.5) |
| ≥80 | 452 (0.6) | 178 (0.6) |

Data are presented as n (% of participants) and mean (standard deviation [SD]).

* Even if previously published, please summarize key model details for reproducibility: deep learning architecture, input size/preprocessing, data augmentation, loss/optimizer, training/validation splits, calibration procedure, and any external validation.

**Response:** Detailed model architecture, preprocessing, training procedures, and validation strategy were fully described in the original RetiAGE development paper and its Supplementary Materials ([Nusinovici et al., Age Ageing 2022](https://academic.oup.com/ageing/article/51/4/afac065/6561972?login=false)). For transparency and reproducibility, we have added a concise summary of the key model components and training parameters to the Methods of the current manuscript, **on page 4-5, line 81-95***: “We previously created a deep learning model utilizing 129,236 retinal images from 40,480 Korean Health Screening study participants. The algorithm was designed to take retinal images from both eye into consideration to lower the possibility of missing out important aging signals. Based on macula-centered retinal photographs, this model was tasked to estimate the likelihood of an individual being 65 years or older. Deep learning algorithm for retinal biological age prediction used a previously developed deep learning model based on the Visual Geometry Group (VGG16) convolutional neural network architecture. Retinal photographs were resized to 300 × 300 pixels and underwent standard data augmentation, including random cropping, rotation, flipping, and photometric adjustments. The network was trained from scratch using the Adam optimizer (learning rate 1 × 10^-4^, batch size 16) and optimized using cross-entropy loss, with model selection based on validation performance. To enhance robustness across ethnicities and imaging conditions, local contrast normalization was applied by subtracting the local mean intensity. Model interpretability was explored using guided backpropagation with SmoothGrad. Full details of model architecture, training procedures, and internal validation have been reported previously.”*

* Given that PIONEER includes individuals >60 years, discuss whether ≥65 remains an optimal threshold and how this affects discrimination/calibration in PIONEER and UK Biobank.

**Response:** RetiAGE outputs a continuous probability reflecting the likelihood that retinal features resemble those of older individuals. This probability scale was applied consistently across cohorts and was analysed as a continuous exposure in all association and prognostic models. Differences in cohort age structure or in the age threshold used during model training do not affect rank-based discrimination, which depends on relative ordering rather than absolute calibration. While alternative age thresholds may influence calibration and the scaling of the output probability, and therefore the magnitude of per-unit effect size estimates, such rescaling does not alter the underlying signal or relative ranking of individuals. Accordingly, the ≥65 reference point does not constrain analyses in either PIONEER or UK Biobank, which serve distinct analytical purposes in this study.

We added a description in the method section on **page 5, line 95-98**: *“RetiAGE was analyzed as a continuous probability score; therefore, the age threshold used during model training affects calibration and coefficient scaling but does not influence rank-based discrimination or association estimates.”*

* The current methods interleave two datasets in a way that is hard to follow. I suggest restructuring into parallel subsections for PIONEER and UK Biobank, each with: participants, imaging protocol, RetiAGE inference, outcomes/definitions, covariates, exclusions, and statistical analysis.

**Response:** We appreciate the suggestion. The Methods section is already organized into parallel descriptions for the PIONEER and UK Biobank cohorts, including participant selection, imaging procedures, outcomes, covariates, and statistical analyses. We have reviewed this section and made minor formatting adjustments to further improve readability.

* When discussing generalizability, explicitly state whether the two cohorts (RetiAGE training cohort vs PIONEER/UKB) are comparable in demographics and imaging protocols, and whether any harmonization or adjustment was performed.

**Response:** We thank the reviewer for this comment. The RetiAGE model was developed in an external Korean cohort and applied unchanged to both PIONEER and UK Biobank. The objective of the current study is not model transportability or domain adaptation, but evaluation of downstream biological and clinical associations within each cohort.

For within-cohort association analyses, comparability of marginal distributions or imaging protocols is not required, provided relevant covariates are adjusted for. Differences in devices or acquisition protocols may shift absolute score distributions but would be expected to introduce non-differential measurement noise, biasing associations toward the null. The consistency of findings across cohorts with substantial demographic and technical differences therefore supports the robustness of the observed associations. We have clarified this scope explicitly in the Discussion and in the limitations **on page 15, line 445-453.**

* Specify the quality-control criteria for fundus images (algorithm, threshold, and performance metrics). Report the proportion excluded and whether excluded participants differ on key variables (age, BMD, comorbidities).

**Response:** Image quality control was applied to ensure that retinal features required for RetiAGE inference were present. Images deemed unreadable were excluded, as they may not contain interpretable retinal information. The quality-control algorithm, threshold, and performance metrics are now described in the Methods **on page 5, line 111-115**, and the proportion of excluded images is reported in the **Supplementary Figure 1**.

* Clarify comparability between the RetiAGE training cohort and PIONEER: are distributions of age, sex, ethnicity, camera type, and dilation similar? Include a short generalizability discussion or sensitivity analyses if there are domain shifts (e.g., different devices/protocols).

**Response:** We thank the reviewer for this comment. We would like to clarify that the objective of this study is not to assess model transportability or to resolve domain gaps between imaging cohorts, but rather to examine the downstream biological and clinical associations of a pre-existing retinal aging biomarker (RetiAGE) across distinct populations.

Importantly, comparability of marginal distributions (e.g., age, ethnicity, camera type, or dilation protocol) is not a prerequisite for valid inference in the context of within-cohort association analyses. Our primary analyses estimate conditional associations between RetiAGE and bone health outcomes after adjustment for relevant covariates within each cohort. Differences in imaging devices or acquisition protocols may shift the absolute distribution of RetiAGE scores, but do not invalidate association estimates derived within a given population.

If present, domain shifts would be expected to introduce non-differential measurement noise in the exposure, biasing effect estimates toward the null rather than generating spurious associations. The observation of consistent cross-sectional and longitudinal associations across cohorts that differ substantially in ethnicity, age distribution, imaging devices, and outcome definitions therefore supports the robustness and biological relevance of the findings rather than undermining them.

We have added a brief clarification in the Discussion to explicitly state that this study focuses on downstream association and risk stratification rather than model generalization or domain adaptation, and that future work will address formal domain harmonization for deployment-oriented applications (**page 15, line 445-453**).

* Please clarify the ophthalmologic disease distribution in both datasets (e.g., diabetic retinopathy, AMD), as these may influence retinal aging estimates.

**Response:** We thank the reviewer for this comment. In the UK Biobank, we have added summaries of potential ophthalmologic conditions that may affect retinal appearance in **Supplementary Table 10**, and conducted sensitivity analyses excluding participants with these conditions; results were consistent with the primary analyses (**Supplementary Table 6**).

In the PIONEER, ophthalmologic conditions were diagnosed as part of the standardized clinical protocol. The prevalence of major blinding eye diseases (e.g., diabetic retinopathy, glaucoma and AMD) are now presented in the **Supplementary Table 10**.

* You use the average of two eyes. Please report the agreement between eyes and, if possible, provide a worst-eye or per-eye sensitivity analysis.

**Response:** Our study focuses on systemic associations rather than eye-specific pathology. Therefore, using RetiAGE scores averaged between the two eyes would better reflect systemic retinal aging.

Furthermore, the RetiAGE scores demonstrated moderate inter-eye agreement (**ICC = 0.65, 95% CI 0.62–0.68**), with a small mean right–left difference (**mean difference = 0.04, SD = 0.23**), indicating no meaningful systematic laterality bias. We have now report this on **page 5, line 115-117**.

* Report the prevalence of retinal abnormalities in PIONEER and any exclusion criteria related to ocular disease that could bias RetiAGE.

**Response:** In the PIONEER, major blinding eye diseases were diagnosed according to a standardized clinical protocol. We have now presented the prevalence of major blinding eye diseases (DR, AMD and glaucoma) in the **Supplementary Table 10**. To address potential bias from the major eye diseases in the current analyses, we performed sensitivity analyses excluding participants with the 3 conditions in either eye and, separately, excluding participants with a history of cataract surgery in the UK Biobank. Results were consistent with the primary analyses (**Supplementary Tables 4, 6 & 7**), supporting the robustness of our findings.

* Provide the distribution of osteoporosis specifically in participants ≥65 years, to align with the RetiAGE threshold.

**Response:** We thank the reviewer for this comment. We have added descriptive analyses illustrating osteoporosis distribution among participants aged ≥65 years in the result section, on **page 11, line 263-264**: “Of the 1,754 participants from PIONEER aged 65 and over, 420 (23.9%) were diagnosed with osteoporosis from DEXA.”

* Consider adding descriptive results for RetiAGE distributions (e.g., mean/SD, quartiles) in each cohort and simple associations with key covariates, to help readers interpret its behavior.

**Response:** We thank the reviewer for this comment. Summary statistics for RetiAGE were presented in Table 1 in the previous manuscript. Additional descriptive analyses and associations with key covariates in both studies are now provided in **Supplementary Table 11 & 12**.

Editorial and consistency issues

* Abstract (lines 12–13): typo “uisng”

* Line 240: “dataset” is strikethrough

* Line 319: typo “propective”

* Supplementary Table: fix “Caucassian” → “White” (and I recommend avoiding the term “Caucasian”). The definition “British, Irish, White, or any other white background” does not correspond to the anthropologic term “Caucasian”; use “White” (as per dataset coding) or the explicit categories. Generalizing the entire British and Irish population as white is not correct.

* References: the citation style shifts around references 3–4.

**Response:** We thank the reviewer for this comment. We have re-checked the manuscript for the typos and reference style issues. We also have changed the “Caucasian” to “White” following the suggestion.

Reviewer 3:

Although this is a secondary implementation of the RetiAGE model, it is still a prediction task where RetiAGE values are used as a predictor for BMD & incident osteoporosis, and thus these warrant detailing of the model's features as per the TRIPOD framework. Below are certain recommendations that will improve the paper significantly:

**Response:** We thank the reviewer for the thoughtful comments. We would like to clarify that this study does not develop or train a new osteoporosis prediction model. Rather, it evaluates the downstream association and prognostic relevance of a previously developed retinal biological aging biomarker (RetiAGE) across independent cohorts. Accordingly, elements of TRIPOD related to model development, training, and internal validation of a new prediction model are not directly applicable. We have nevertheless strengthened reporting of data processing, model application, and performance metrics where appropriate.

1. No mention of Outliers: There is no mention of removal of outliers or the methods used for identification and removal.

**Response:** Data quality control was performed prior to analysis, as described in the Method section. Retinal images used for model training underwent manual review by trained graders to exclude unreadable images ([Nusinovici et al., Age Ageing 2022](https://academic.oup.com/ageing/article/51/4/afac065/6561972?login=false)). DEXA outputs were screened for implausible or non-numeric values indicative of data entry or extraction errors (e.g., string entries in numeric fields). Values consistent with physiological ranges were retained. We have clarified these data quality control procedures in the Methods section, on **page 5, line 126-128**.

2. No internal/external validation of an osteoporosis prediction model.

**Response:** RetiAGE was developed and internally validated in the Korean Health Screening Study, as previously reported. In the present study, RetiAGE was applied without retraining to two independent cohorts (PIONEER and UK Biobank) for evaluation of downstream associations and prognostic relevance with bone-related outcomes. As no new prediction model was trained, internal or external validation in the TRIPOD sense does not apply. We have clarified this distinction in the Methods section.

3. No Github code/ supplementary material required for reproducibility.

**Response:** The source code for the RetiAGE model is publicly available and was released with the original development publication ([Nusinovici et al., Age Ageing 2022](https://academic.oup.com/ageing/article/51/4/afac065/6561972?login=false). <https://github.com/medi-whale/UKBIOBANK_FUNDUS_Classifier>). The current study applies this pre-trained model to independent datasets and does not introduce new model training code.

4. PIONEER dataset is not publicly available.

**Response:** PIONEER is an ongoing population-based cohort study. In accordance with institutional and ethical requirements, individual-level data cannot be publicly released but can be made available to qualified researchers upon reasonable request and completion of a data-sharing agreement. This statement has been added to the Data Availability section.

5. No discrimination/calibration/ interpretability metrics used.

**Response:** We thank the reviewer for this suggestion. As this study evaluates the prognostic relevance of RetiAGE rather than training a new osteoporosis prediction model, performance metrics were selected according to study design. In PIONEER, we report measures of association and added model fitness for continuous bone outcomes. In UK Biobank, time-to-event discrimination was assessed using the concordance index (C-index). To explore potential incremental value, we additionally evaluated risk reclassification. These analyses have been clarified in the Methods and Results sections. Interpretability analyses of the RetiAGE model itself were previously reported in the original publication.
